# Properties and Synergistic Mechanism of Ultra-High-Performance Concrete Incorporating Spontaneous Combustion Gangue (Sand) and Phosphorus Slag

**DOI:** 10.3390/ma19102079

**Published:** 2026-05-15

**Authors:** Yannian Zhang, Youlin Ye, Yingliang Tan, Qiyue Ren, Wande Li, Tingyi Yan, Qingjie Wang, Qi Wu

**Affiliations:** 1 School of Transportation Engineering, Dalian Jiaotong University, Dalian 116028, China; 2 School of Transportation and Geomatics Engineering, Shenyang Jianzhu University, Shenyang 110168, China; 3 Meishan Yichuan Construction Co., Ltd., Meishan 620500, China; 4 Meishan Huantian Industrial Park Investment and Operation Co., Ltd., Meishan 620500, China; 5 School of Civil Engineering, Shenyang Jianzhu University, Shenyang 110168, China

**Keywords:** eco-UHPC, industrial solid waste, chloride resistance, microstructure, synergistic utilization

## Abstract

**Highlights:**

- PS substitution improves UHPC flowability by up to 16.83%.
- Substitution of 40% SCGS optimizes particle packing density and aggregate gradation.
- Persistent pozzolanic reactions generate C-S-H gel, enhancing late-stage compressive strength.
- Consuming CH produces C-S-H gel, creating a dense and uniform microstructure.
- Superior chloride penetration resistance was achieved with PS content below 20%.

**Abstract:**

The sustainable application of ultra-high-performance concrete (UHPC) is often constrained by high material costs and environmental footprints. While the individual effects of various industrial wastes have been extensively studied, the synergistic mechanism of multi-source waste in UHPC remains poorly understood. To fill the research gap, an eco-UHPC was developed wherein river sand (RS) was partially replaced by spontaneous combustion gangue sand (SCGS), and Portland cement (PC) was partially replaced by spontaneous combustion gangue (SCG) powder and phosphorous slag (PS). A systematic investigation was conducted to assess the packing density, flowability, mechanical properties, chloride ion penetration resistance, and micromorphology. The results indicate that 40% SCGS substitution (by mass) optimizes particle packing density and aggregate gradation, while PS incorporation significantly improves flowability by up to 16.83%. Notably, persistent pozzolanic reactions and the consumption of Ca(OH)_2_ facilitate the generation of dense C-S-H gel, which creates a uniform microstructure and enhances late-stage compressive strength. Furthermore, superior chloride penetration resistance is achieved when the PS content is maintained below 20%. These findings support the synergistic utilization of SCGS, SCG, and PS in UHPC production, while facilitating broader application of UHPC through reduced costs and lower carbon emissions.

## 1. Introduction

Ultra-high-performance concrete (UHPC), composed primarily of silica fume (SF), Portland cement (PC), fine aggregates, and steel fibers, is characterized by superior mechanical properties and durability. Consequently, it has become a critical material for major infrastructure projects such as cross-sea bridges and long tunnels. However, the widespread application of UHPC is restricted by high production costs, a substantial carbon footprint due to high cement usage, and the scarcity of river sand (RS) and expensive quartz sand. Simultaneously, the global accumulation of industrial solid waste presents a significant environmental challenge. In China, over 3.8 billion tons of new industrial solid waste was generated in 2023. The disposal and comprehensive utilization of such waste have become urgent global concerns. To address the issues of high costs, carbon emissions, and aggregate shortages, extensive research has turned towards the partial replacement of cement and aggregates with solid waste [1,2,3,4]. This approach is increasingly recognized as a vital pathway for the sustainable development of UHPC and the high-value utilization of solid waste.

In recent years, a growing body of literature has investigated the use of solid wastes, such as spontaneous combustion gangue (SCG), fly ash, slag powder, rice husk ash, municipal solid waste incineration fly ash, and recycled concrete powder (RCP), to mitigate the environmental impact of UHPC [5,6,7]. For instance, Bai et al. [8] developed a low-carbon UHPC utilizing the micro-filling and pozzolanic effects of graphite tailings powder. This modification reduced the carbon footprint by 106.71 kg CO_2_/m^3^ and cement usage by 208.34 kg/m^3^ while maintaining mechanical performance. Liu et al. [9] found that replacing 25–50% of the quartz powder with finely ground graphite tailings can comprehensively improve mechanical properties, durability, environmental performance, and economic value. Similarly, Wu et al. [10] demonstrated that partially replacing PC with RCP alleviates environmental pressure. However, it was noted that RCP may negatively impact resistance to chloride ion (Cl^−^) penetration [11]. Yonis et al. [12] found that thermo-mechanically treated RCP influenced the workability of UHPC. Furthermore, studies on phosphorous slag (PS) indicate that while PS may reduce early-age strength, PS significantly enhances flowability and long-term strength [13]. Although slag powder has been shown to improve resistance to Cl^−^ penetration, it is associated with increased drying shrinkage under high-temperature conditions [14]. Significant advances have also been made regarding aggregate replacement. Sun et al. [15] used graded gold tailing sand as the sole aggregate to prepare eco-friendly UHPC. Wang et al. [16] utilized expanded perlite as a substitute for quartz sand; while workability remained largely unaffected, a marked decrease in compressive strength was observed. Liu et al. [17] validated the feasibility of using waste foundry sand, reporting satisfactory strength in UHPC beams containing this alternative aggregate. Nguyen et al. [18] found that calcined bauxite aggregate enhances fire resistance and mitigates the severity of explosive spalling. Additionally, investigations by Zhang et al. [19] into the macro-performance and microstructure of UHPC containing recycled concrete sand (RCS) confirmed its potential as an RS substitute. However, it has been consistently reported that the inclusion of RCS reduces flowability [19,20,21], with performance deteriorating further as the particle size of RCS decreases [22].

Overall, these studies highlight the potential of solid waste in UHPC production. However, existing research has largely focused on the single substitution of either cement or aggregates [13,23]. Such single-substitution approaches often compromise specific performance aspects, thereby limiting the maximum utilization rate of waste materials and hindering significant reductions in the carbon footprint. Although the feasibility of using multiple solid wastes has been preliminarily explored [24,25,26], the synergistic mechanisms remain poorly understood and require further investigation. To address this gap, this study proposes a multi-waste synergistic approach utilizing SCG, spontaneous combustion gangue sand (SCGS), and PS to achieve complementary physical and chemical benefits. During the crushing of SCG, particles of varying sizes were produced. Fine powders serve as cement replacements, while coarser particles (SCGS) replace river sand. SCG has been shown to refine the hydration process [27] and enhance both compressive strength and Cl^−^ impermeability [28]. SCGS serves as an RS replacement while providing internal curing effects. Concurrently, the slow but sustained pozzolanic reaction of PS generates C-S-H gel during mid-to-late curing stages, compensating for the reduced strength gain typically observed in later cement hydration. Furthermore, the combined pozzolanic activity of SCG and PS is expected to consume CH, thereby optimizing the microstructure.

Building upon prior research by our group [28], this study aims to develop an eco-friendly UHPC by replacing 20% of PC with SCG powder, 0–30% of PC with PS, and 0–60% of RS with SCGS. Mechanical property tests were conducted to determine the optimal SCGS content. Subsequently, fresh-state properties, compressive strength, hydration products, and microstructure were characterized to validate the effectiveness of the SCG(S)-PS system for eco-UHPC. The effects of varying PS contents on workability and microstructure were investigated, and the synergistic mechanisms between SCG and PS, as well as between solid waste and PC, were elucidated. The findings presented in this study provide a theoretical basis for the efficient utilization of solid waste, offering significant value for the engineering application and promotion of eco-UHPC.

## 2. Materials and Methods

### 2.1. Test Methods

#### 2.1.1. Wet Packing Density

The methods in the literature [28,29] were used to calculate the wet packing density of UHPC. Three sections of the churned slurry were poured into a volume V mold. Until the mold was completely filled, the slurry was poured to fill less than one-third of the mold height and vibrated thirty times. After wiping away any extra paste, the mass was noted. The wet packing density was then determined using the computational technique [28]. The average value of the three experimental measures was used to determine the outcome.

#### 2.1.2. Fluidity

The flow table test method outlined in the Chinese standard GB/T 2419-2005 [30] was used to determine the fluidity of UHPC. After removing any extra mortar from the tabletop, the freshly mixed UHPC was poured into the mold in two layers. The flow table was then instantly turned on after the standard mold was raised vertically. The diameter was measured in two mutually perpendicular directions after 25 vibrations were ensured, and the average of the diameters was used to determine the fluidity of UHPC.

#### 2.1.3. Setting Time

The setting time of UHPC was determined using a Vicat apparatus (Wuxi Zhongke Building Materials Instrument Co., Ltd., Wuxi, China) in compliance with the Chinese standard GB/T 1346-2011 [31]. The Vicat needle was initially brought into light contact with the surface of freshly made UHPC, held still for 1–2 s, and then allowed to enter the mixture. When further penetration stopped and the distance between the needle tip and the base plate settled within 4 ± 1 mm, the initial setting time was recorded. The mold was then inverted, and all timing began when the water addition was finished. The total setting time was determined when the needle stopped leaving a circular impression on the UHPC surface.

#### 2.1.4. Compressive Strength

In compliance with EN 196-1 [32], the compressive strength of UHPC was measured using prismatic specimens measuring 40 mm × 40 mm × 160 mm. At each curing interval (3, 7, 28, and 60 days), six specimens were evaluated and the average of the six readings was used to determine the representative compressive strength of UHPC. In addition, the loading rate during testing was 2 mm/min.

#### 2.1.5. XRD

X-ray diffraction (XRD) was employed to characterize the phase composition of UHPC. Samples were taken from the center of the specimens after the compression test, soaked in anhydrous ethanol for five days to stop hydration, subsequently dried, and ground to powder. Testing was conducted on a Bruker D8 Advance system (Cu Kα, λ = 1.54 Å) operating at 45 kV and 40 mA. Data collection covered a scanning range of 5–90° at a rate of 5°/min.

#### 2.1.6. SEM

Scanning electron microscopy (SEM) on a ZEISS Gemini 300 instrument (Carl Zeiss Microscopy Deutschland GmbH, Jena, Germany) was used to examine the microstructure of UHPC specimens that had been cured for 28 days. Samples were taken from the center of the specimens after the compression test.

#### 2.1.7. Rapid Chloride Ion Migration Test (RCMT)

The resistance of UHPC to chloride ingress was evaluated in accordance with NT Build 492 [33]. Cylindrical specimens (Φ100 × 50 mm) were first subjected to a vacuum for 3 h, followed by immersion in a CH solution for 1 h under vacuum, and a further soaking period of 18 ± 2 h at atmospheric pressure. Upon completion of the test, the cylinders were split open and treated with AgNO_3_ to reveal the depth of chloride penetration. Data were collected from 10 points per specimen, with 3 specimens per group, and the average values were used to derive the non-steady-state migration coefficient (D_RCM_) via the method in the literature [28].

#### 2.1.8. Capillary Water Absorption

ASTM C1585-13 [34] was utilized to determine the capillary water absorption. Cylindrical specimens (Φ100 × 50 mm) were first subjected to oven drying at 50 ± 2 °C for a duration of 3 days. To prevent evaporation, epoxy resin was used, while the top surface was covered with a waterproof film. The samples were then positioned in a reservoir where the water level was maintained between 3 and 5 mm above the base of the specimen. Mass measurements were recorded at specific intervals during the initial 6 h. Prior to each weighing, surface moisture was blotted using a dry towel, and the specimen was placed on the balance with the non-immersed face pointing upwards. The final reported value was derived from the average of three specimens.

#### 2.1.9. Ecological Evaluation

The life cycle assessment (LCA) of UHPC mixtures containing various dosages of SCGS and PS was assessed by five indexes in compliance with EN ISO 14040 [35] and EN ISO 14044 [36]. The ecological evaluation indexes are calculated by summing the energy consumption or emissions of all raw materials, whereas inventory data for the remaining constituents were retrieved from previous studies [28,37,38]. As industrial by-products, both SCGS and PS can be regarded as zero-emission materials. Moreover, to access the effective utilization of raw materials, the carbon intensity (*Ci*) was also calculated, which represents CO_2_ emissions per unit of performance obtained [39].

### 2.2. Materials

**Figure 1:**
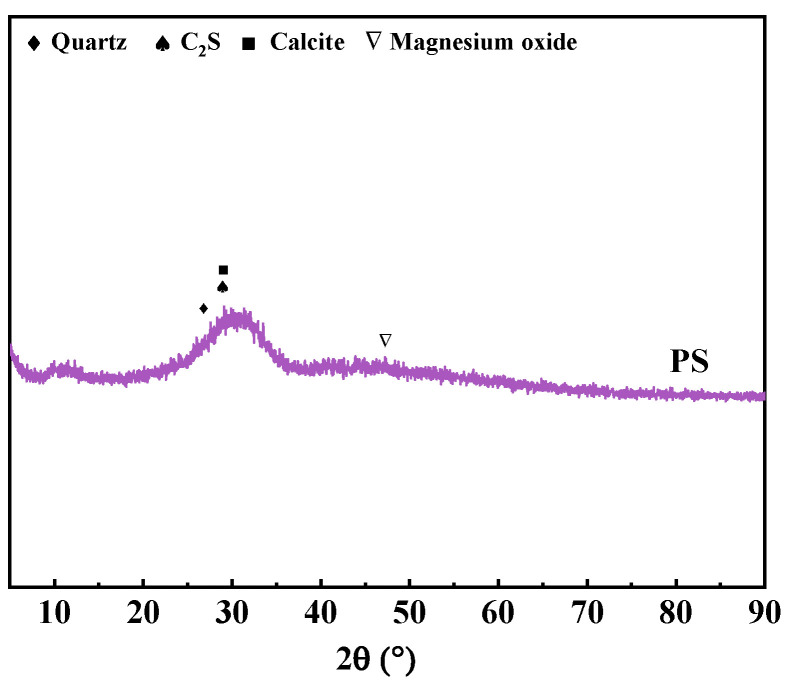
XRD pattern of PS.

**Figure 2:**
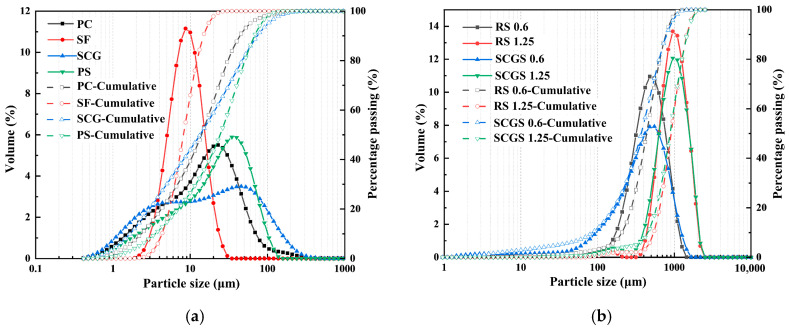
The particle size distribution of raw materials: (**a**) cementitious materials; (**b**) aggregate.

**Figure 3:**
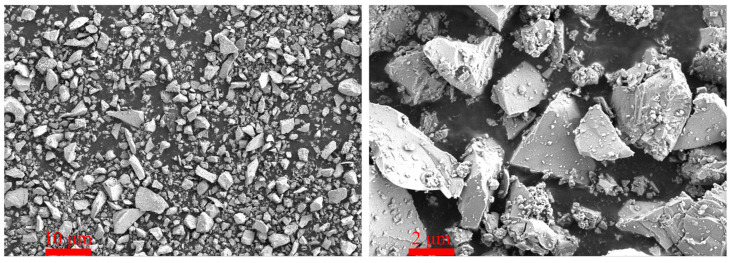
SEM images of PS.

The cementitious materials employed in this study included P·I 52.5 Portland cement (PC), silica fume, SCG, and PS. RS and SCGS served as the fine aggregates, and both were classified into two particle size ranges: 0.075–0.6 mm and 0.6–1.25 mm. The PS was sourced from Kunming, Yunnan Province, China. The chemical composition of the raw materials and their particle size distribution were determined using a PANalytical Axios X-ray fluorescence spectrometer (PANalytical B.V., Almelo, The Netherlands) and a Malvern Mastersizer 2000 laser particle size analyzer (Malvern Instruments Ltd., Malvern, UK), respectively. The specific surface area was obtained from nitrogen adsorption measurements at 77 K on a Micromeritics ASAP 2460 instrument (Micromeritics Instrument Corporation, Norcross, GA, USA), and calculated using the BET method after proper vacuum degassing of the dried samples. The pore size distribution was also measured by Micromeritics ASAP 2460. The chemical compositions and physical properties of PS are detailed in Table 1, the specific surface area of PS is 0.66 m^2^/g, and the chemical compositions and physical properties of PC, SF, and SCG are shown in a previous investigation by our research group [28]. The X-ray diffraction (XRD) pattern of the PS is presented in Figure 1. The XRD pattern of PS exhibits an amorphous halo between 25 and 35° 2θ, which is attributed to the retention of a glassy structure in PS due to the insufficient time for silicate melt to crystallize during the rapid cooling process of waste residue generated from the production of yellow phosphorus. These amorphous phases constitute the primary source of the potential hydraulic activity and pozzolanic activity of PS. The PS utilized in this study primarily consists of amorphous phases and minor crystalline phases, such as calcite, quartz, and magnesium oxide, which is similar to the results measured in other studies [40,41,42]. Figure 2 illustrates the particle size distributions of the raw materials. It can be observed that the particle size of PS is coarser than that of both PC and SCG, whereas the SCGS exhibits a finer gradation compared to the RS. The micromorphology of the PS was examined via scanning electron microscopy (SEM), as shown in Figure 3. The images reveal that the PS particles are characterized by an irregular and angular shape. A polycarboxylate-based superplasticizer (SP) was utilized to regulate the workability of the UHPC mixtures.

### 2.3. Preparation of UHPC

**Figure 4:**
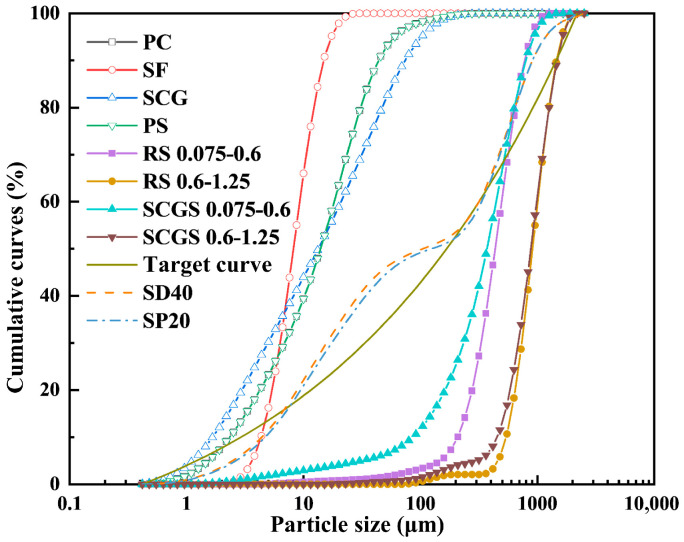
Raw material particle size distribution, target curve, and optimized gradation curves.

**Figure 5:**
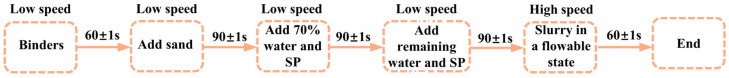
Mixing process design for UHPC.

Drawing upon previous investigations by our research group [28], the mixture designated S20 was selected as the control. The experimental program was conducted in two phases. Firstly, RS was replaced by SCGS at increments of 20%, 40%, and 60% (by mass). Following the identification of the optimal SCGS content, PS was introduced to replace PC at ratios of 10%, 20%, and 30%, resulting in a total cement substitution of 30–50%. The mixtures incorporating 20–60% SCGS were designated as SD20-SD60, while those containing 10–30% PS were labeled SP10-SP30. The mix proportions were designed using the modified Andreasen & Andersen particle packing model [43,44]. The target and optimized particle size distribution curves are illustrated in Figure 4, and the detailed mix compositions are listed in Table 2. Additionally, the mixing protocol adopted for this study is depicted in Figure 5. The mixing equipment is a JJ-5 planetary cement mortar mixer, operated in manual mode. During low-speed mixing, the rotation and revolution speeds are 140 ± 5 r/min and 62 ± 5 r/min, respectively; during high-speed mixing, the rotation and revolution speeds are 285 ± 10 r/min and 125 ± 10 r/min, respectively. All specimens were maintained under standard curing conditions, with the temperature controlled at 20 ± 1 °C and relative humidity kept at ≥95%.

## 3. Results and Discussion

### 3.1. The Effect of SCGS on the Performance of UHPC

#### 3.1.1. Wet Packing Density

**Figure 6:**
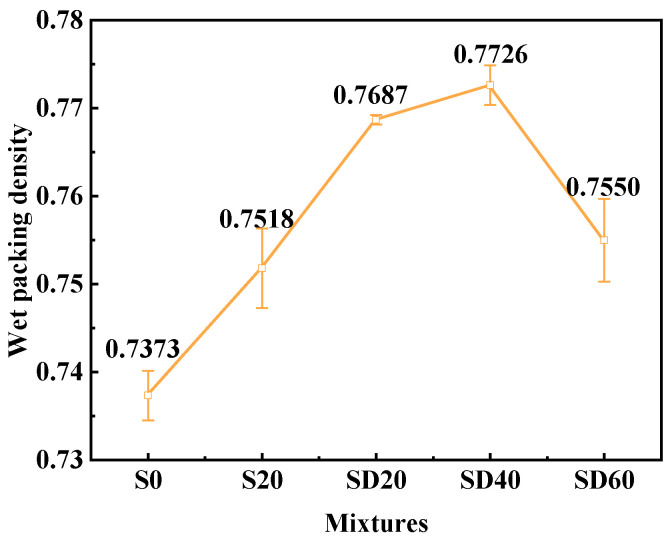
Wet packing density of UHPC with different SCGS contents.

Figure 6 presents the wet packing density test results of UHPC with varying SCGS contents. As shown in the figure, the packing density initially increased with the SCGS content and reached a maximum value of 0.7726 in the SD40 group, after which it decreased. This initial increase resulted from the finer particle size of SCGS compared with RS. Specifically, the D50 values of the two SCGS grades (370.700 μm and 880.101 μm) were lower than those of RS (435.645 μm and 908.638 μm), enabling a filler effect that promoted the formation of a dense packing system [45,46]. Accordingly, the addition of SCGS improved the aggregate gradation, filled interstitial pores, and increased the packing density. In contrast, when the SCGS content increased to 60%, the packing density declined. This decline primarily resulted from the adverse influence of excessive SCGS on UHPC gradation, coupled with the high water absorption of SCGS, which reduced free water content and led to air entrapment, expansion, and increased interparticle spacing. Nevertheless, all mixtures with SCGS exhibited higher packing densities than the S0 group, demonstrating that SCGS positively influenced the packing density.

#### 3.1.2. Fluidity

**Figure 7:**
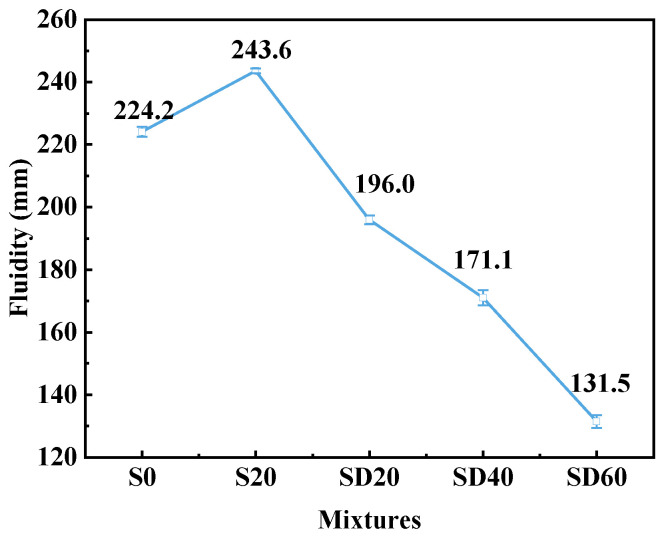
Fluidity of UHPC with different SCGS contents.

Figure 7 presents the fluidity test results of UHPC mixtures prepared with different contents of SCGS. In contrast to the fluidity-enhancing effect typically associated with SCG, the incorporation of SCGS led to a notable reduction in fluidity. Relative to the S20 group, the flowability values of the SD20, SD40, and SD60 groups decreased by 19.54%, 29.76%, and 46.02%, respectively. This decline in fluidity is primarily attributable to the physical characteristics of SCGS. This decline is primarily due to the angular shape of SCGS particles, as shown in Figure 3, which increases interparticle friction compared to the smooth, rounded RS [2]. Additionally, the porous, moisture-retentive microstructure of SCGS absorbs free water, reducing lubrication and further decreasing fluidity.

#### 3.1.3. Compressive Strength

**Figure 8:**
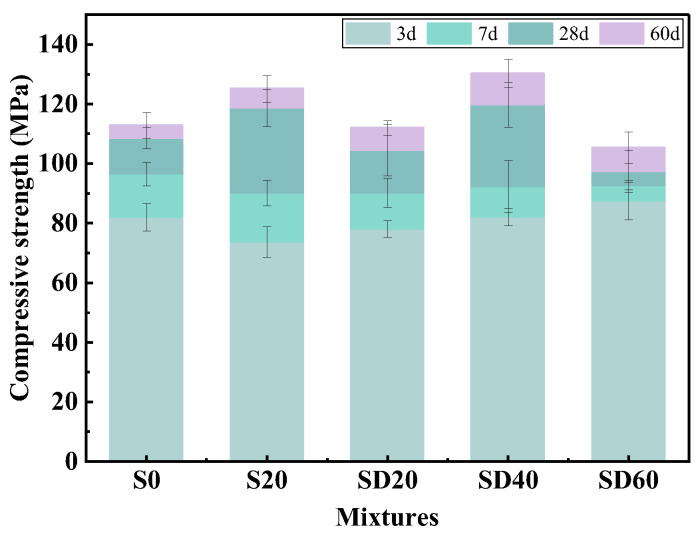
Compressive strength of UHPC with different SCGS contents.

Figure 8 shows the compressive strength development of UHPC mixtures incorporating varying contents of SCGS. At 3 days of curing, the compressive strength of the SD20, SD40, and SD60 groups increased by 5.97%, 11.39%, and 18.58%, respectively, compared to the S20 group. In contrast to the adverse effect typically observed with SCG on early-age strength, SCGS promoted early strength development with the SD60 group even exceeding the S0 group. This improvement is primarily due to the water absorption characteristic of SCGS, which effectively lowered the actual water–binder ratio and thereby enhanced early strength. By 7 days, the rate of strength growth in SCGS-modified mixtures slowed. Compared to the S20 group, the growth rates for the SCGS-modified groups were 0.03%, 2.44%, and 2.86%, respectively.

After 28 days, a distinctly different strength development pattern emerged. The SD40 mixture achieved the highest compressive strength among all groups, reaching 119.78 MPa at 28 days and further increasing to 130.24 MPa at 60 days. In contrast, the other SCGS-modified mixtures exhibited lower strength values than the S20 group at these ages. The superior performance of the SD40 group is primarily ascribed to the internal curing effect provided by SCGS. Similar to other porous materials [47], SCGS gradually releases previously absorbed moisture during the later stages of cement hydration, promoting continued hydration and resulting in enhanced microstructural densification and strength. In contrast, the SD60 group, which contained the highest SCGS content, exhibited excessive water absorption. This led to air entrapment during vibratory compaction, forming a greater number of large voids and consequently diminishing the final compressive strength.

#### 3.1.4. SCGS Optimal Dosage Determination

Based on the above experimental results, the maximum values for compressive strength and wet packing density are achieved at 40% SCGS content. Although fluidity gradually decreases with increasing SCGS content, it still achieves a value of 171.1 mm at 40% SCGS, indicating satisfactory workability. Therefore, the SD40 group with 40% SCGS has been selected as the optimal group for subsequent experiments.

### 3.2. The Effect of PS on the Performance of UHPC

Based on the SD40 (containing 20% SCG and 40% SCGS), cement was further replaced with PS at contents ranging from 10% to 30% to evaluate its influence on UHPC performance.

#### 3.2.1. Wet Packing Density

**Figure 9:**
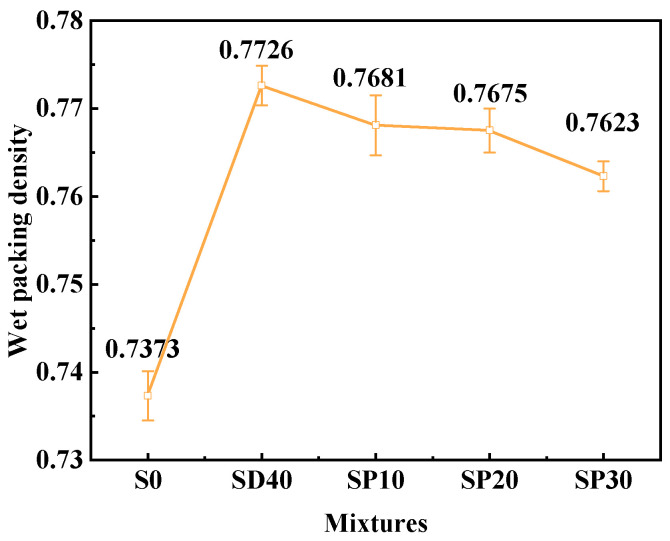
Wet packing density of UHPC with different PS contents.

Figure 9 presents the wet packing density results of UHPC with varying PS contents. Compared to the SD40 group, the SP10, SP20 and SP30 groups exhibited reductions in packing density of 0.58%, 0.66% and 1.33%, respectively. There was a downward trend in wet packing density as the PS content increased. Previous analysis indicates that increased free water content reduces air interlocking and enhances packing density. Although the incorporation of PS increased the free water content, a decline in wet packing density was still observed. This phenomenon is primarily due to the larger particle size of the PS (D50 = 24.38 μm). The increased content of large-particle PS disrupts the compact packing framework of UHPC, thereby reducing packing density. It is worth noting that although the addition of PS led to a reduction in wet packing density, the positive effect of SCGS on particle packing was sufficient to compensate for the adverse influence of PS. Consequently, the wet packing density of UHPC prepared using SCG(S)-PS consistently exceeded that of the solid waste-free group by ≥3.39%. Furthermore, compared to the sample S20 in a previous study [28], which solely used SCG to replace 20% PC, the wet packing density of SP20 (0.7675) was 2.10% higher than that of S20 (0.7518) under conditions where SCGS replaced 40% of the RS, SCG replaced 20% of the PC, and PS replaced 20% of the PC. This confirms that the synergistic effect of multiple solid waste components has a positive impact on the wet packing density of UHPC.

#### 3.2.2. Fluidity

**Figure 10:**
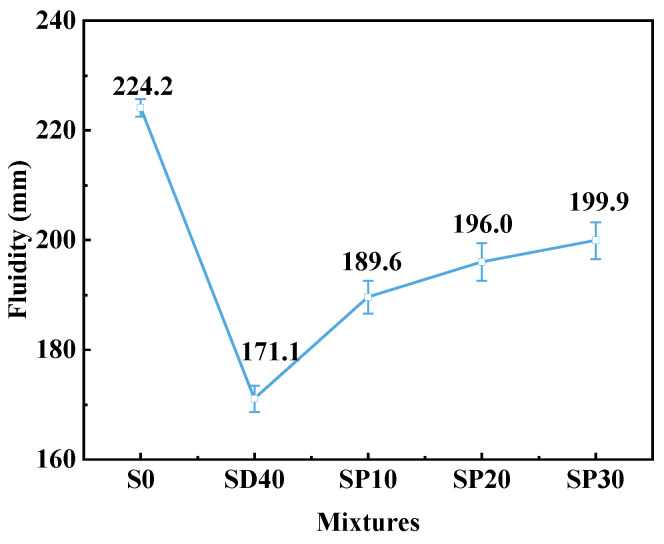
Fluidity of UHPC with different PS contents.

Figure 10 shows the fluidity test results of UHPC mixtures incorporating varying PS replacement levels. As the PS replacement ratio for PC increases, it can be seen that the fluidity of UHPC gradually increases. Compared to the SD40 group, the fluidity of the SP10, SP20 and SP30 groups increased by 10.81%, 14.55% and 16.83% respectively. This improvement in fluidity can be mainly attributed to the increased effective water–cement ratio resulting from cement replacement by PS. The reduced cement content diminishes ion release upon hydration, weakening electrostatic interactions between cement particles and enhancing the dilution effect, which collectively improve fluidity [45,46,48]. A further contributing factor is the smaller specific surface area of PS (0.66 m^2^/g) compared to cement (1.12 m^2^/g). The incorporation of PS reduces the overall specific surface area of the binder system, thereby lowering water demand and consequently enhancing flow performance. Furthermore, compared to S20 in a previous study [28], the fluidity of SP20 decreased from 243.6 mm to 196 mm. This is because the substitution of 40% RS with SCGS in SD40 reduced the fluidity to 171.1 mm. Overall, the incorporation of PS significantly improves the fluidity of eco-UHPC. Moreover, the fluidity of SP20 remains above 180 mm [3,49], facilitating its application in practical construction projects.

#### 3.2.3. Setting Time

**Figure 11:**
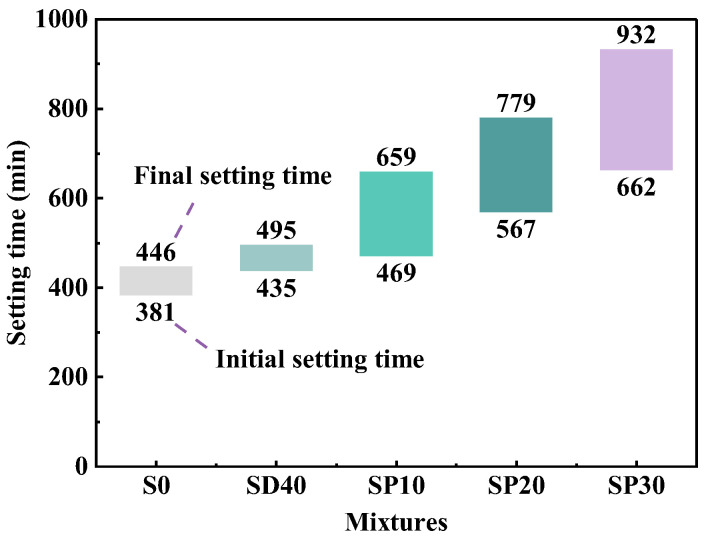
The setting time of UHPC with different PS contents.

Figure 11 shows the results of the setting time test for UHPC at different PS content levels. As can be seen, the setting time gradually increases with rising PS content. Compared to the SD40 group, the initial setting times of the SP10, SP20, and SP30 groups increased by 7.82%, 30.34%, and 52.18%, respectively, and the final setting times increased by 33.13%, 57.37%, and 88.28%, respectively. It should be noted that the tested specimens were prepared as pure paste without aggregates, which explains the identical results observed between the SD40 and S20 groups. This consistency also applies to subsequent XRD and SEM analyses conducted under the same conditions.

The pronounced retardation effect induced by PS incorporation can be attributed to both its dilution effect on cement and the chemical influence of residual soluble phosphorus and fluorine compounds. The presence of phosphorus pentoxide (P_2_O_5_) in PS reduces the alkalinity of the cementitious system and promotes the formation of insoluble calcium phosphate (Ca_3_(PO_4_)_2_) layers on cement particle surfaces [50], which hinders normal hydration reactions. Furthermore, the phosphorus and fluorine elements in PS react with Ca^2+^ ions in the solutions, delaying the crystallization of CH [51] and consequently extending the setting time. These findings demonstrate that PS effectively retards early-age hydration reactions even under the low water–binder ratio conditions characteristic of UHPC systems.

#### 3.2.4. Compressive Strength

**Figure 12:**
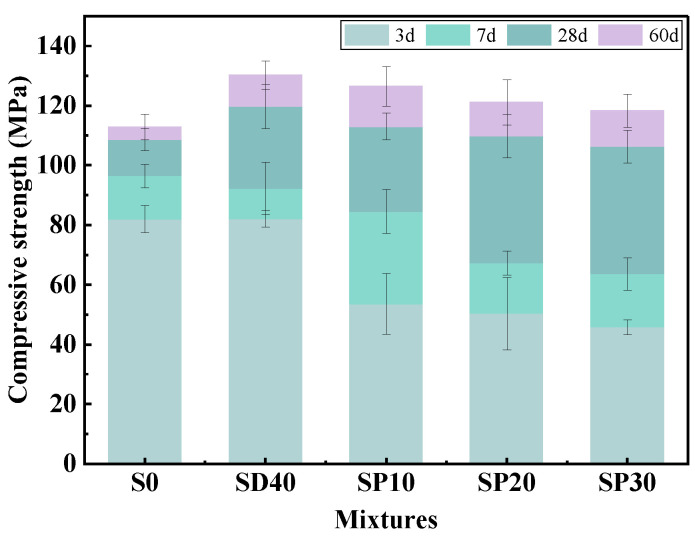
Compressive strength of UHPC with different PS contents.

Figure 12 shows the compressive strength test results of UHPC at different PS contents. At 3 days, the compressive strength of UHPC decreased significantly. Compared to the SD40 group, the reductions were 34.77%, 38.67%, and 44.25% for SP10, SP20, and SP30, respectively. These results are consistent with those observed in other PS-containing UHPC [13,23,52]. This confirms the retarding effect of PS, whereby the formation of hydration products is reduced and consequently compressive strength is lowered. At 7 days, the adverse effects of PS on compressive strength decreased to 8.36%, 27.10%, and 31.06%, respectively.

As the curing age progressed, a notable enhancement in compressive strength was observed at 28 days. Compared to the SD40 group, the 28-day compressive strengths of the SP10-SP30 groups were reduced by 5.59%, 8.35%, and 11.19%, respectively. Although these values remained lower than that of the SD40 group, the difference was markedly reduced. Furthermore, the compressive strengths of the SP10 and SP20 groups exceeded that of the S0 group, with the SP10 group achieving the highest value of 113.0 MPa. Even the SP30 mixture, which exhibited the lowest compressive strength among PS-modified groups, reached 106.3 MPa, a level nearly equivalent to that of the S0 group. Furthermore, a significant recovery in 28-day compressive strength was evident, with the extent of recovery increasing as the PS content rose. This is due to the continuous breakdown of the surface inert layer of PS in the strongly alkaline environment provided by CH. During the later stages of hydration, PS demonstrated appreciable pozzolanic activity, contributing to the formation of additional C-S-H gel, which enhanced the microstructure and thereby substantially improved the 28-day compressive strength of UHPC. It is noteworthy that the solid waste-free control group (S0) exhibited only marginal strength gain by 60 days, whereas the specimens incorporating solid waste, especially those with PS, continued to develop considerable strength. At 60 days, the compressive strengths of the SP10 to SP30 groups surpassed that of the S0 group by 12.10%, 7.36%, and 4.89%, respectively. The SP10 group attained the maximum value of 126.5 MPa, demonstrating that the compressive strength of eco-UHPC prepared using SCG(S)-PS can exceed that of the solid waste-free UHPC. In addition, compared to S20 in a previous study [28], the 28-day compressive strength of SP20 decreased by 7.48%, but remained higher than that of the control group S0 without solid waste. This indicates that when 40% of RS is replaced with SCGS, 20% of PC is replaced with SCG, and 20% of PC is replaced with PS, the prepared eco-UHPC not only exhibits satisfactory compressive strength [53] but also achieves substantial and high-value utilization of solid waste.

**Figure 13:**
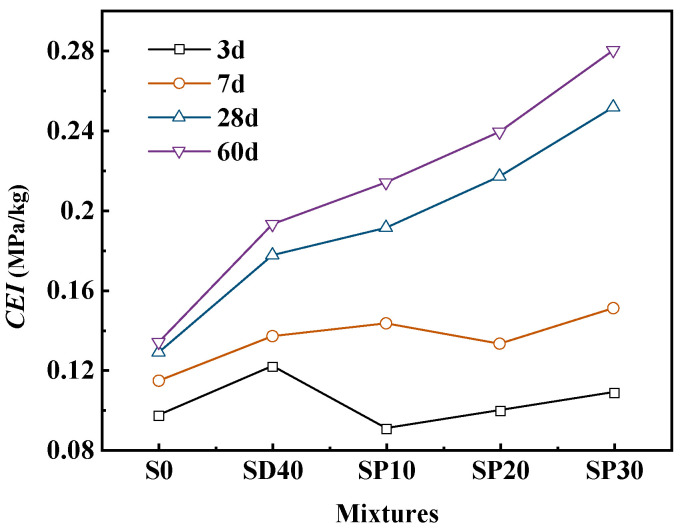
The cement efficiency index of UHPC with different PS contents.

To investigate the impact of PS on PC utilization efficiency, the PC efficiency index (*CEI*) was employed for assessment [54,55], as per Equation (1), with the results presented in Figure 13.

(1) $$CEI=f_{c}/C$$

In the equation, *f_c_* represents the compressive strength of UHPC at different curing ages (MPa), and *C* represents cement content per m^3^(kg).

As shown in Figure 13, the addition of PS reduced the *CEI* values at 3 days and 7 days, with the *CEI* value of the SP10 group even falling below that of the S0 group. This suggests that PS has an adverse effect on early compressive strength. However, at 28 and 60 days, the *CEI* values increased significantly, demonstrating that PS has a pronounced positive effect on the late-stage compressive strength of UHPC. Replacing PC with PS can significantly enhance the utilization efficiency of PC.

#### 3.2.5. XRD

**Figure 14:**
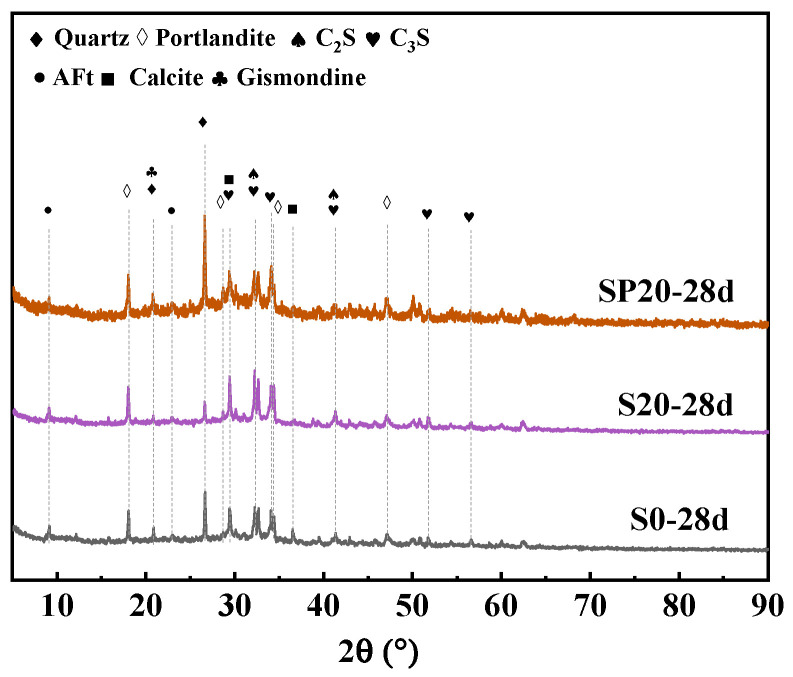
XRD patterns of UHPC with different PS contents at 28 days.

As illustrated in Figure 14, the XRD test results of UHPC at varying PS contents are presented. The incorporation of PS did not result in any alteration to the types of hydration products present in UHPC. The primary phases consist of CH (PDF#44-1481), AFt (PDF#41-1451), calcite (PDF#05-0586), and cement clinker mineral phases. Compared to S20, in the SP20 group, as the PC replacement rate reached 40%, the C_2_S (PDF#33-0302) and C_3_S (PDF#49-0442) peaks exhibited a certain degree of decrease, indicating a reduction in the number of unhydrated cement particles. Compared to S0, the C_2_S and C_3_S peaks in SP20 show a slight increase, which is attributed to the inhibitory effect of PS on the hydration reaction. Furthermore, the SiO_2_ content of PS is significantly higher than that of PC, resulting in a markedly higher quartz peak in the SP20 compared to the S0 group.

#### 3.2.6. SEM

**Figure 15:**
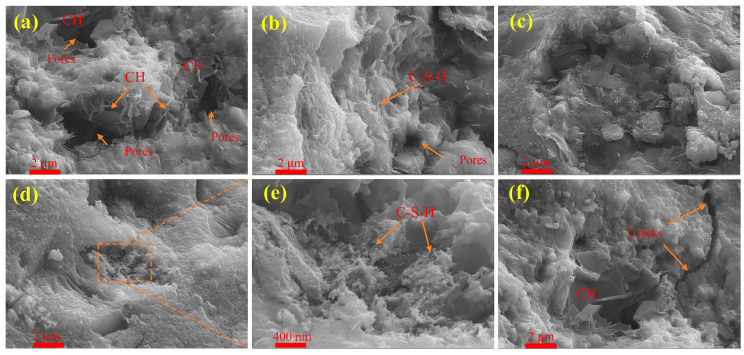
SEM images of UHPC with different PS contents at 28 days: (**a**) S0 group; (**b**) SD40 group; (**c**) SP10 group; (**d**) SP20 group; (**e**) SP20 group local magnification images; (**f**) SP30 group.

Figure 15 presents SEM images of UHPC at 28 days with varying PS contents. The main hydration products observed include plate-like CH and C-S-H gel. Microstructural analysis indicates that all PS-modified mixtures exhibit a denser matrix compared to the solid waste-free group, accounting for the enhanced mechanical properties of PS-containing UHPC. As shown in Figure 15a, the reference group without solid waste displays an abundance of CH, a phase characterized by low intrinsic strength and weak interfacial bonding, representing a vulnerable component in the microstructure. Magnification of the orange rectangular region in Figure 15d, presented in Figure 15e, reveals that larger pores are filled with flocculent C-S-H gel. This indicates that, during later hydration, PS acts synergistically with SCG, exhibiting pozzolanic reactivity that converts CH into higher-strength C-S-H gel, thereby refining the microstructure. Furthermore, the favorable workability of eco-UHPC promotes uniform dispersion of unreactive particles, which serve as heterogeneous nucleation sites for C-S-H formation [56]. This process enhances C-S-H formation, filling interparticle voids and contributing to a more compact microstructure. However, as evident in Figure 15f, notable microcracks are present in mixtures with high PS content. Excessive PS incorporation strongly retards early-age hydration, considerably reducing hydration product formation and increasing interparticle spacing. Therefore, the PS replacement level in this system should not exceed 20% to ensure microstructural integrity and sustained strength development.

#### 3.2.7. RCMT

**Figure 16:**
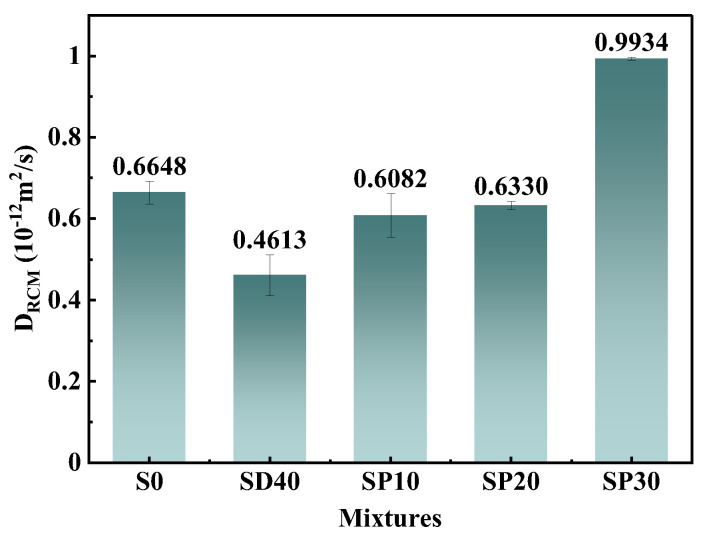
RCMT results of UHPC with different PS contents at 28 days.

Figure 16 presents the RCMT results for UHPC with different PS contents. Compared to the SD40 group, the DRCM values of SP10-SP30 groups increased by 31.84%, 37.22%, and 115.35%, respectively. The lower reactivity of PS compared to PC reduced hydration product formation with increasing PS content, thereby decreasing UHPC density and consequently increasing DRCM values, which adversely affected chloride ion resistance. Nevertheless, as previously discussed, the high pozzolanic activity of SCG and the nucleation effect of inert particles from PS and SCGS largely compensated for this drawback by densifying the matrix and blocking connected pores [11,57]. Consequently, the prepared eco-UHPC showed lower DRCM values than the solid waste-free group at PS contents ≤20%. Moreover, the SP30 group (with 40% RS replacement and 50% PC replacement: SCG20% + PS30%) achieved a D_RCM_ value of 0.9934 × 10^−12^ m^2^/s, substantially below the 2.5 × 10^−12^ m^2^/s threshold. These results demonstrate that SCG(S)-PS-prepared eco-UHPC possessed exceptional resistance to Cl^−^ permeation.

#### 3.2.8. Capillary Water Absorption

**Figure 17:**
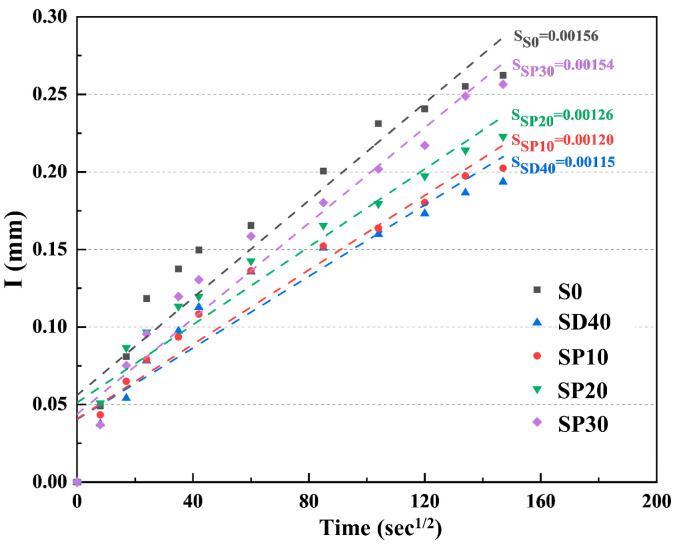
The capillary water absorption of UHPC at 28 days under different PS contents.

Figure 17 presents the capillary water absorption test results for UHPC specimens with different PS contents at 28 days. Relative to the SD40 group, the water absorption coefficients of the SP10, SP20, and SP30 groups increased by 4.35%, 9.57%, and 33.91%, respectively, indicating that PS incorporation adversely affects the water resistance of UHPC. Notably, the SD40 group had an adsorption coefficient that was 26.28% lower than the S0 group, which was the lowest among all groups. This enhanced performance primarily stems from three mechanisms. Firstly, as detailed in Section 3.1, the finer particle size of SCGS improves packing density and reduces water absorption [58]. Secondly, SCGS participates in secondary hydration reactions, generating additional C-S-H gel [59] that blocks water penetration pathways. Thirdly, the porous structure of SCGS creates an internal curing effect that promotes PC hydration and increases hydration product formation. This reduces capillary pores and enhances water resistance. It is worth emphasizing that the eco-UHPC prepared using the SCGS-PS synergistic technology consistently demonstrates superior water resistance performance compared to the conventional UHPC containing no solid waste.

#### 3.2.9. Ecological Evaluation

**Figure 18:**
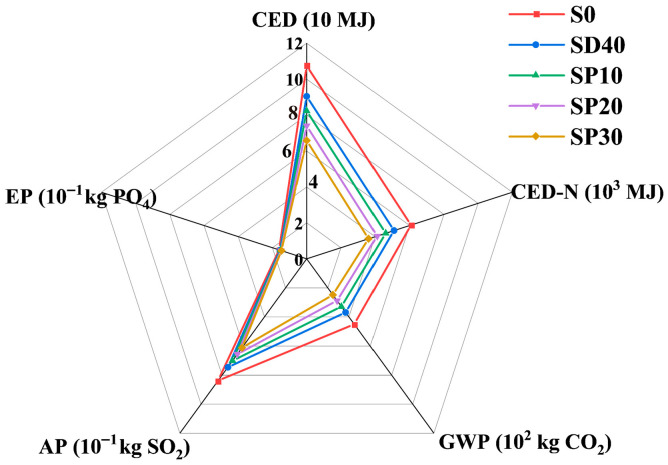
Ecological assessment of 1 m^3^ UHPC with different PS contents.

Figure 18 displays the ecological assessment results for 1 m^3^ of UHPC mixtures with different PS contents. As shown in Figure 18, both energy consumption and adverse factor emissions decrease significantly across all groups with increasing PS content. Compared to the S0 group, the SP30 group achieved a 38.69% reduction in primary renewable energy input (CED), a 40.44% decrease in primary non-renewable energy input (CED-N), a 45.07% reduction in global warming potential (GWP), a 27.06% decrease in acidification potential (AP), and an 8.70% reduction in eutrophication potential (EP).

**Figure 19:**
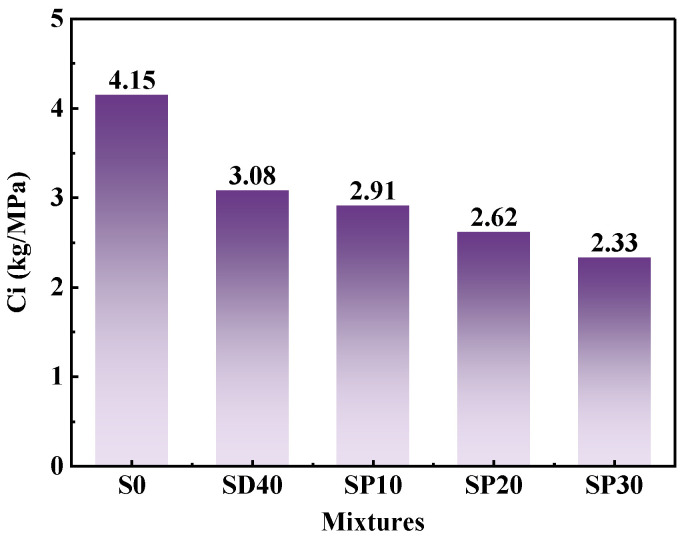
Carbon intensity of 1 m^3^ UHPC with different PS contents.

In addition, Figure 19 shows the carbon intensity (*Ci*) values of UHPC with varying PS contents. Compared to the S0 group, the *Ci* values decreased by 25.75%, 29.92%, 36.83%, and 43.89%, respectively. This progressive reduction in *Ci* values indicates that the incorporation of SCG(S) and PS results in reduced CO_2_ emissions during UHPC production, thereby improving cement utilization efficiency. Consequently, the synergistic use of SCG(S) and PS facilitates the development of UHPC that combines outstanding engineering properties with improved ecological sustainability.

## 4. Conclusions

In this study, an eco-UHPC was developed through the co-utilization of SCGS, SCG, and PS, and their synergistic effects on packing density, flowability, mechanical properties, and chloride resistance were systematically investigated. It was revealed that the synergistic utilization of SCGS, SCG, and PS can optimize microstructure, enhance late-stage compressive strength, and ensure excellent resistance to chloride ion penetration, while reducing material costs and carbon emissions. The following conclusions can be drawn:

(1) The pozzolanic activity of PS is weaker than that of SCG, but partial substitution of PC with PS can significantly improve the fluidity of the system and ensure uniform distribution of various phases. The fluidity of UHPC gradually increases with the increase in PS content, with a maximum improvement of up to 16.83% at a replacement rate of 30%. The pozzolanic activity of PS and SCG is utilized, leading to the generation of more C-S-H gel, which fills the pores and enhances the compactness of eco-UHPC. This results in a synergistic advantage of the pozzolanic effect and fluidity regulation effect of SCG and PS.
(2) The inert particles of SCGS and PS effectively improve the aggregate gradation of UHPC, which benefits the formation of a dense packing system and improves the wet packing density of UHPC. When the content of SCGS reaches 40%, the wet packing density of UHPC achieves its maximum value (0.7726). The excellent fluidity enables the inert particles to disperse evenly, forming good heterogeneous crystal nucleus points. This facilitates rapid filling of interstitial voids between inert particles by C-S-H gels, resulting in a dense microstructure.
(3) The SiO_2_ reactivity of both SCG and PS is found to be relatively low. However, the strongly alkaline environment provided by CH can penetrate the surface inert layer, thereby enabling continuous SiO_2_ hydration. The pozzolanic reaction in SCG and PS is characterized by its slowness and persistence, with a marked acceleration occurring during the mid-to-late stages (7–60 days) to continuously generate C-S-H gel. This approach successfully compensates for the cement’s natural reduction in strength development during the final hydration phase. As a result, compressive strengths exceeding those of UHPC without solid waste at 60 days are achieved.
(4) The UHPC without solid waste generates more CH, which is the weak link of the system due to its low strength and weak inter-crystal bonding. The pozzolanic reaction of SCG and PS consumes a large amount of CH, which transforms the low-strength CH into high-strength C-S-H gel. In particular, a certain number of capillary pores and interfacial transition zone pores are formed at the early stage of cement hydration, and the C-S-H gel generated by the pozzolanic reaction can aim to fill these pores, making the microstructure of UHPC dense and uniform, which is an important contributor to why the compressive strength of the system is significantly higher than that of UHPC without solid waste.
(5) The weaker activity of PS leads to a slight decrease in the Cl^-^ permeability resistance of UHPC. However, SCG exhibits higher pozzolanic reaction activity, and PS exerts an efficient modification effect on fluidity, particularly with regard to the efficient nucleation effect of uniformly dispersed inert particles of PS and SCGS. In this case, C-S-H gels grow interspersed with inert particles to form a reticulated intertwined dense structure, which effectively divides the originally connected pore network into disconnected isolated small pores. The connected pores that make Cl^-^ diffuse or permeate rapidly are completely solved, so the system shows very high chloride ion penetration resistance, and especially when the PS doping is not more than 20%, the chloride ion penetration resistance of the eco-UHPC is better than that of the group without solid waste.

In this study, a systematic investigation was conducted to assess the packing density, flowability, mechanical properties, chloride ion penetration resistance, and micromorphology. In the future, we will conduct an in-depth analysis of eco-UHPC, including EDS, TG/DTG, MIP, quantitative XRD, and CH consumption analysis. Furthermore, although incorporating solid waste into UHPC can reduce costs and lower the carbon footprint, practical application still faces numerous challenges, such as the diversity of solid waste types and variations in chemical composition even among waste with the same designation. In the future, in-depth research in this area is needed to enhance the scalability of solid waste systems.

## Figures and Tables

**Table 1:** Chemical composition and physical properties of PS (wt. %).

SiO_2_	Al_2_O_3_	CaO	Fe_2_O_3_	MgO	SO_3_	K_2_O	Na_2_O	TiO_2_	P_2_O_5_	LOI	D10 (μm)	D50 (μm)	D90 (μm)
32.31	2.54	56.39	0.46	2.42	0.94	0.66	0.28	0.19	2.29	–	3.51	24.38	67.71

**Table 2:** Mix proportion of UHPC (kg/m^3^).

Mixtures	PC	SCG	PS	SF	RS_0–0.6_	RS_0.6–1.25_	SCGS_0–0.6_	SCGS_0.6–1.25_	Water	SP
S0	844	0	0	169	709	304	0	0	182	20
S20	675	169	0	169	709	304	0	0	182	20
SD20	675	169	0	169	567	243	142	61	182	20
SD40	675	169	0	169	425	182	284	122	182	20
SD60	675	169	0	169	284	122	425	182	182	20
SP10	591	169	84	169	425	182	284	122	182	20
SP20	506	169	169	169	425	182	284	122	182	20
SP30	422	169	253	169	425	182	284	122	182	20

## Data Availability

The original contributions presented in this study are included in the article. Further inquiries can be directed to the corresponding author.

## References

[1] Liu Y., Wei Y. (2021). Effect of calcined bauxite powder or aggregate on the shrinkage properties of UHPC. Cem. Concr. Compos..

[2] Lv Y., Qin Y., Wang J., Li G., Zhang P., Liao D., Xi Z., Yang L. (2022). Effect of incorporating hematite on the properties of Ultra-High Performance Concrete including nuclear radiation resistance. Constr. Build. Mater..

[3] Shi J., Pan W., Kang J., Yu Z., Sun G., Li J., Zheng K., Shen J. (2024). Properties of Ultra-High Performance Concrete incorporating iron tailings powder and iron tailings sand. J. Build. Eng..

[4] Zhang Y., Liu W., Wang Q., Zhang L. (2025). Coupling mechanisms and synergistic effects in Portland Cement-ceramic powder-ground granulated blast furnace slag-CFB desulfurization ash composite binder. Waste Biomass Valorization.

[5] Sun C., Yan C., Li J., Ma J., Li Y. (2025). Ultra-high-performance concrete from 100% solid waste: Mechanical strength, microstructure and sustainability analysis. Case Stud. Constr. Mater..

[6] Zhao X., Nematollahi B., Chougan M., Xiao J. (2025). Approaches to reduce cost and environmental impacts of UHPC production: A review. Case Stud. Constr. Mater..

[7] Song J., Banthia N., Yoo D.-Y. (2025). Effect of supplementary cementitious materials on durability of Ultra-High-Performance Concrete: A review. Cem. Concr. Compos..

[8] Bai Q., Xin Z., Ma H.-Y., Sun W.-H., Liu H.-B., Jiang C.-J. (2025). Graphite tailings powder driving the low-carbon transformation of Ultra-High Performance Concrete (UHPC): Innovative pathways for solid waste resource utilization and synergistic mechanisms for performance enhancement. Constr. Build. Mater..

[9] Liu H.-B., Xin Z., Ma H.-Y., Guo H.-Y., Bai Q., Jiang C.-J. (2026). Utilization of finely ground graphite tailings as a sustainable substitute for quartz powder in ultra-high performance concrete: A comprehensive study on performance, microstructure, and environmental impact. J. Build. Eng..

[10] Wu H., Liu C., Zhao Y., Chen G., Gao J. (2024). Elucidating the role of recycled concrete powder in low-carbon Ultra-High Performance Concrete (UHPC): Multi-performance evaluation. Constr. Build. Mater..

[11] Sun C., Chen L., Xiao J., Singh A., Zeng J. (2021). Compound utilization of construction and industrial waste as cementitious recycled powder in mortar. Resour. Conserv. Recycl..

[12] Yonis A., Oinam Y., Pyo S. (2026). Hydration, fresh and mechanical properties of clinker-free ultra-high performance concrete (UHPC) incorporating thermo-mechanically activated waste concrete powder. J. Build. Eng..

[13] Yang R., Yu R., Shui Z., Gao X., Xiao X., Zhang X., Wang Y., He Y. (2019). Low carbon design of an Ultra-High Performance Concrete (UHPC) incorporating phosphorous slag. J. Clean. Prod..

[14] Yalçınkaya Ç., Yazıcı H. (2017). Effects of ambient temperature and relative humidity on early-age shrinkage of UHPC with high-volume mineral admixtures. Constr. Build. Mater..

[15] Sun Z., Wang Y., Ji T., Zhang Y., Fang Y., Li L., Liu J., Hu Z. (2026). Gold tailings sand to prepare eco-friendly ultra-high performance concrete matrix: A synergistic approach to fine aggregate-cementitious material packing density for performance enhancement. Case Stud. Constr. Mater..

[16] Wang X., Wu D., Geng Q., Hou D., Wang M., Li L., Wang P., Chen D., Sun Z. (2021). Characterization of sustainable Ultra-High Performance Concrete (UHPC) including expanded perlite. Constr. Build. Mater..

[17] Liu S., Zheng W., Wu F. (2023). Preparation of ultra-high performance concrete containing waste foundry sand and its application in structures. Structures.

[18] Nguyen N.-V., Ngo T.T., Thai D.-K., Kim S.-E. (2026). Enhancing fire resistance and mitigating spalling in non-fibrous UHPC using calcined bauxite aggregate. J. Build. Eng..

[19] Zhang H., Ji T., Zeng X., Yang Z., Lin X., Liang Y. (2018). Mechanical behavior of Ultra-High Performance Concrete (UHPC) using recycled fine aggregate cured under different conditions and the mechanism based on integrated microstructural parameters. Constr. Build. Mater..

[20] Chen K., Cheng S., Wu Q., Chen X., Zhao C., Li S., Lu J. (2024). Utilization of recycled fine aggregate in Ultra-High Performance Concrete: Mechanical strength, microstructure and environment impacts. Constr. Build. Mater..

[21] Zhou Y., Guo D., Xing F., Guo M. (2021). Multiscale mechanical characteristics of Ultra-High Performance Concrete incorporating different particle size ranges of recycled fine aggregate. Constr. Build. Mater..

[22] Guo Y., Gao D., Qin D., Pi H. (2025). Properties of UHPC with totally recycled fine aggregates and its mixture design method. J. Build. Eng..

[23] Chen G., Huang Y., Yang R., Yu R., Xiao R., Wang Z., Ke X., Xie G., Cheng J., Bao M. (2023). Comparative study on mechanical properties and microstructure development of Ultra-High Performance Concrete incorporating phosphorous slag under different curing regimes. Constr. Build. Mater..

[24] Xu P., Liu G., Wang Y., Zhu Y., Wan X., Xu H. (2025). Properties of UHPC products prepared by sawing mud and gold mine tailings via static pressure forming. Case Stud. Constr. Mater..

[25] Du J., Zou K., Chen R., Zhou J., Wang X., Zou Y., Zhang Z., Yang J. (2026). A low-clinker design of ultra-high-performance concrete (UHPC) with high-volume slag activated by flue gas desulfurization (FGD) gypsum. Constr. Build. Mater..

[26] Zhu M., Xue S., Zhu J., Ahmad M.R., Dai J.-G. (2026). Development and characterization of underwater-cast ultra-high-performance geopolymer concrete. Constr. Build. Mater..

[27] Li Z., Gu X., Liu B., Liu J., Zhang Y., Yang B., Cheng B., Kong Y., Nehdi M.L., Zhang L. (2024). A novel approach for revealing the strength evolution mechanism of limestone-calcined clay cement with self-ignition coal gangue and shell powder. Constr. Build. Mater..

[28] Xu Y., Wu Q., Zhang Y., Han D., Nehdi M.L., Zhang L. (2024). Preparation of Ultra-High Performance Concrete using spontaneous combustion gangue as a substitute for cement. Mater. Today Commun..

[29] Kwan A.K.H., Ng P.L., Huen K.Y. (2014). Effects of fines content on packing density of fine aggregate in concrete. Constr. Build. Mater..

[30] (2005). Test Method for Fluidity of Cement Mortar.

[31] (2011). Test Methods for Water Requirement of Normal Consistency, Setting Time and Soundness of the Portland Cement.

[32] (2005). Methods of Testing Cement: Determination of Strength.

[33] (1999). Concrete, Mortar and Cement-Based Repair Materials: Chloride Migration Coefficient from Non-Steady-State Migration Experiments.

[34] (2013). Standard Test Method for Measurement of Rate of Absorption of Water by Hydraulic Cement Concrete.

[35] (1997). Environmental Management-Life Cycle Assessment-Principles and Framework.

[36] (2006). Environmental Management-Life Cycle Assessment-Requirements and Guidelines.

[37] Wang X., Yu R., Shui Z., Song Q., Liu Z., Bao M., Liu Z., Wu S. (2019). Optimized treatment of recycled construction and demolition waste in developing sustainable Ultra-High Performance Concrete. J. Clean. Prod..

[38] Müller H.S., Haist M., Vogel M. (2014). Assessment of the sustainability potential of concrete and concrete structures considering their environmental impact, performance and lifetime. Constr. Build. Mater..

[39] Damineli B.L., Kemeid F.M., Aguiar P.S., John V.M. (2010). Measuring the eco-efficiency of cement use. Cem. Concr. Compos..

[40] Wang L., Guo F., Lin Y., Yang H., Tang S.W. (2020). Comparison between the effects of phosphorous slag and fly ash on the C-S-H structure, long-term hydration heat and volume deformation of cement-based materials. Constr. Build. Mater..

[41] Zhang Y., Wu Q., Yang D., Wang Q., Qu Z., Zhong Y. (2024). Study on the properties of alkali-activated phosphorus slag mortar mixed with granulated blast furnace slag/fly ash. J. Aust. Ceram. Soc..

[42] Zhang Y., Yang D., Wang Q. (2023). Performance study of alkali-activated phosphate slag-granulated blast furnace slag composites: Effect of the granulated blast furnace slag content. Arch. Civ. Mech. Eng..

[43] Yu R., Spiesz P., Brouwers H.J.H. (2014). Mix design and properties assessment of Ultra-High Performance Fibre Reinforced Concrete (UHPFRC). Cem. Concr. Res..

[44] Yu R., Spiesz P., Brouwers H.J.H. (2015). Development of an eco-friendly Ultra-High Performance Concrete (UHPC) with efficient cement and mineral admixtures uses. Cem. Concr. Compos..

[45] Wang D., Shi C., Farzadnia N., Shi Z., Jia H. (2018). A review on effects of limestone powder on the properties of concrete. Constr. Build. Mater..

[46] Fan D., Yu R., Shui Z., Wu C., Wang J., Su Q. (2020). A novel approach for developing a green Ultra-High Performance Concrete (UHPC) with advanced particles packing meso-structure. Constr. Build. Mater..

[47] Wang X., Yu R., Shui Z., Song Q., Zhang Z. (2017). Mix design and characteristics evaluation of an eco-friendly Ultra-High Performance Concrete incorporating recycled coral based materials. J. Clean. Prod..

[48] Fan D., Zhang C., Lu J., Liu K., Yin T., Dong E., Yu R. (2023). Recycling of steel slag powder in green Ultra-High Strength Concrete (UHSC) mortar at various curing conditions. J. Build. Eng..

[49] Zhu P., Du S., Heng P., Zhang L., Zhang S., Wu Y. (2025). Investigation on Mix Proportions of Ultra-High Performance Concrete with Recycled Powder and Recycled Sand. Buildings.

[50] Lee N.K., Lee H.K. (2013). Setting and mechanical properties of alkali-activated fly ash/slag concrete manufactured at room temperature. Constr. Build. Mater..

[51] Su Y., Zhao H., He X., Zheng Z., Ma Q., Ding J., Bao M. (2023). The effect of wet-grinding phosphorus slag on the hydration kinetics of Portland cement. Constr. Build. Mater..

[52] Huang Y., Chen G., Yang R., Yu R., Xiao R., Wang Z., Xie G., Cheng J. (2023). Hydration kinetics and microstructure development of Ultra-High Performance Concrete (UHPC) by high volume of phosphorus slag powder. Cem. Concr. Compos..

[53] (2015). Reactive Powder Concrete.

[54] Fan D., Yu R., Shui Z., Liu K., Feng Y., Wang S., Li K., Tan J., He Y. (2021). A new development of eco-friendly Ultra-High Performance Concrete (UHPC): Towards efficient steel slag application and multi-objective optimization. Constr. Build. Mater..

[55] Fan D., Yu R., Fu S., Yue L., Wu C., Shui Z., Liu K., Song Q., Sun M., Jiang C. (2021). Precise design and characteristics prediction of Ultra-High Performance Concrete (UHPC) based on artificial intelligence techniques. Cem. Concr. Compos..

[56] Zhang Z., Wang Q., Yang J. (2017). Hydration mechanisms of composite binders containing phosphorus slag at different temperatures. Constr. Build. Mater..

[57] Sun C., Chen L., Xiao J., Liu Q., Zuo J. (2021). Low-carbon and fundamental properties of eco-efficient mortar with recycled powders. Materials.

[58] Ahmed T., Elchalakani M., Karrech A., Mohamed Ali M.S., Guo L. (2021). Development of eco-UHPC with very-low-C3A cement and ground granulated blast-furnace slag. Constr. Build. Mater..

[59] Duan X., Xia J., Yang J. (2014). Influence of coal gangue fine aggregate on microstructure of cement mortar and its action mechanism. J. Build. Mater..

